# Is comorbidity alone responsible for changes in health-related quality of life among critical care survivors? A purpose-specific review

**DOI:** 10.1186/s13054-024-04997-x

**Published:** 2024-06-26

**Authors:** Lotti Orwelius, Susanne Wilhelms, Folke Sjöberg

**Affiliations:** 1 Department of Anaesthesia and Intensive Care, Linköping University Hospital, 581 85 Linköping, Sweden; 2 Department of Biomedical and Clinical Sciences, Linköping University, 581 83 Linköping, Sweden; 3 Department of Medical and Health Sciences, Faculty of Health Sciences, Linköping University, 581 83 Linköping, Sweden; 4 Burns, Hand, and Plastic Surgery, Linköping University Hospital, 581 85 Linköping, Sweden; 5 Department of Clinical Physiology, Faculty of Medicine, Linköping University, 581 83 Linköping, Sweden

**Keywords:** Critical care, Intensive care unit, Health-related quality of life, Outcome

## Abstract

**Background:**

Health-related quality of life (HRQoL) is one of the most important outcome variables for assessing the effectiveness of intensive care, together with mortality and survival, where comorbidity is suggested to have high impact. However, studies are lacking that examine to what extent HRQoL is affected after a general ICU period, beyond that of the effects that may be claimed to be due to comorbidities.

**Design:**

Purpose-specific literature review including literature searches in PubMed, Cinahl, Scopus, and Cochrane library between 2010 and 2021.

**Measurements and results:**

This Purpose-specific, i.e., task focused review examines HRQoL (assessed by either SF-36 or EQ-5D, > 30 days after leaving the hospital) in adult patients (≥ 18 years) having an ICU length of stay > 24 h. Further, the HRQoL comparisons were adjusted for age or comorbidity. A total of 11 publications were found. A majority comprised observational, prospective cohort studies, except three that were either case–control, cross-sectional comparison, or retrospective cohort studies. A total of 18,566 critically ill patients were included, and the response rate ranged from 16 to 94%. In all studies, a recurrent relevant finding was that HRQoL after ICU care was affected by pre-ICU comorbidities. In three studies (n = 3), which included a comorbidity adjusted control group, there were no effect of the critical care period itself on the registered HRQoL after the critical care period.

**Conclusion:**

Health-Related Quality of Life (HRQoL) in former ICU patients appears to be primarily influenced by comorbidity. A notable limitation in this field of research is the high heterogeneity observed in the studies reviewed, particularly in terms of the HRQoL measurement tool employed, the duration of follow-up, the methodology for comorbidity assessment, and the adjustments for age and sex. Despite these variations and the limited number of studies in the review, the findings suggest a minimal HRQoL impact beyond the effects of comorbidity. Given the significant dearth of comprehensive studies in this domain, there is an escalating call for more thorough and detailed research endeavours.

**Graphical abstract:**

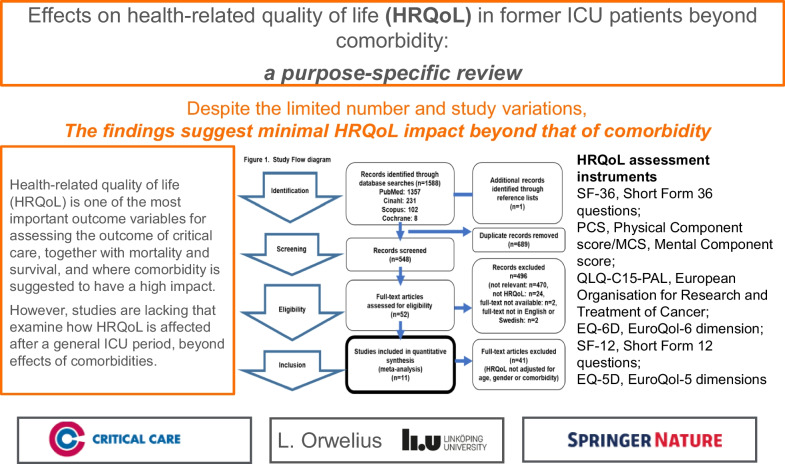

**Supplementary Information:**

The online version contains supplementary material available at 10.1186/s13054-024-04997-x.

## Introduction

Measuring outcome after critical illness is important for many reasons, and historically, mortality has been used particularly as a measure of the effectiveness of intensive care treatment [1]. However, the development of new medical procedures for critical care patients has led to increased survival despite more complex illnesses and extensive injuries. Therefore, the need for outcome measures other than survival has been claimed to be increasingly important [2]. Furthermore, the long-term patients’ perspective has in parallel been considered increasingly relevant in evaluating long-term post-ICU treatment outcomes. As a consequence, health-related quality of life (HRQoL) has gained its place as one of the most important outcome variables for assessing the effectiveness of intensive care, together with mortality and survival [3].

It is well known that HRQoL is reduced after intensive care compared with that in the general population [4]. However, a recurring issue is that ICU patients are compared with a healthy population, most often not properly adjusted for effects of comorbidities. In other HRQoL assessments comorbidities are a well-known, and probably the most important, factor for the perceived quality of life described by the individual [5–7]. In particular, up to 73% of patients admitted to the ICU have developed one or more chronic comorbidity in the years before admission to the ICU [8, 9]. In addition, a considerable proportion, approximately 15%, are diagnosed with a new “chronic” or long-term health condition at the event which has led to the critical care period [8]. Adding this together, a very large proportion, approximately 80–90%, of ICU patients have comorbidities [8, 9]. One confounding consideration for this issue is that, not seldomly, adjustment for age is claimed to be a substitute for comorbidities, because there is a well-known collinearity between the two [4]. Another comorbidity adjustment that needs to be mentioned is the Charlson comorbidity index. However, the Charlson comorbidity index is not an HRQoL adjustment score but a mortality prediction score with a low HRQoL sensitivity for most diseases other than the most lethal [10].

With a high proportion of comorbidities in the ICU cohorts, it may be argued that the decrease in the level of HRQoL seen post-ICU may already be present in the period before intensive care. It is then concomitantly uncertain when, and if, the pre-ICU HRQoL level is reached. Most of the published data so far suggest that HRQoL levels seem to reach a plateau at approximately one year after the ICU period [4]. This temporal relationship has just recently been extensively reviewed [11]. In most studies, the level of HRQoL in ICU patients is lower than in the control groups, which might be related to control groups that have not been fully adjusted for comorbidities.

Comorbidities, by definition, hold paramount significance in any context when assessing HRQoL. Consequently, in evaluating HRQoL among these individuals, it is imperative to account for the impact of pre-existing comorbidities. The objective of this targeted review is therefore to meticulously evaluate the extent to which current published research on HRQoL post-critical care has been adequately adjusted for the elevated comorbidity levels in former ICU patients.

## Methods

This is a purpose specific review targeting the extent to which current published research on HRQoL post-critical care has adequately been adjusted for the elevated comorbidity levels in former ICU patients [12]. The choice of the specific review type, “purpose specific review” was made to target specifically the issue of comorbidity effects on post ICU HRQoL.

### Data sources and search strategy

**Fig. 1:**
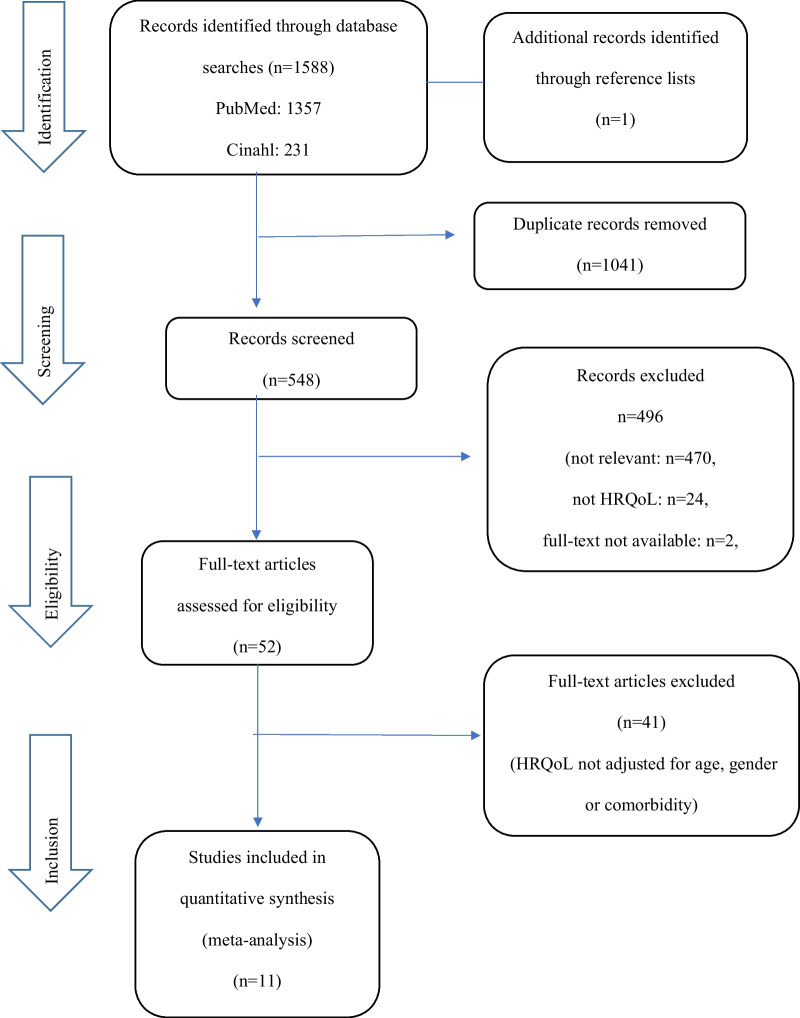
PRISMA study flow diagram

This purpose-specific systematic review was written in accordance with the Equator network PRISMA-statement and conducted according to the PICO (Population, Intervention, Controls Outcome) strategy to ensure a systematic search of the available literature. The search method used to identify all relevant articles was developed and discussed by two authors (LO and FS), and the final string was approved by all authors. An electronic search strategy was developed in collaboration with a librarian with extensive experience in systematic reviews. The process was done in three steps. Firstly, the literature searches were conducted in PubMed, Cinahl, Scopus, and the Cochrane library independently by two reviewers (LO and SW), who eliminated clearly irrelevant articles based on title, abstract, and full-text levels (Fig. 1). During this screening process a third author (FS) was involved and any disagreements were settled by a consensus process. The following terms were used, with all terms mapped to the appropriate MeSH term/equivalent function using the medical subject headings: for Population (P); (“intensive care unit” OR “critical care” OR “critical illness”) AND (“intensive care unit” OR “ICU”): for Intervention (I); (“follow-up studies” OR “aftercare”); Controls (C) is not applicable for this review: for Outcome (O); (“health-related quality of life” OR “HRQoL” OR “QoL”) AND (“physical ability”) OR (“cognitive dysfunction”) OR “ADL” OR mortality. Full-text articles published in peer-reviewed journals were considered for inclusion. Reference lists of retrieved articles and relevant publications of expert authors were screened to identify additional papers that met the inclusion criteria.

Applied inclusion criteria were; articles evaluating HRQoL after intensive care, published in the period of January 1, 2010, to December 31, 2021, adult patients (≥ 18 years), and treated at a general intensive care unit. The search period from 2010 was chosen due to catch the newest research in the field. Articles describing paediatric follow up and follow up after specialised intensive care treatment such as burn unit, cardiothoracic- or neuro-intensive care were excluded. Articles were also excluded if they described a subgroup of the general ICU population regarding diagnoses, treatment, sex, or specific age groups. In addition, articles were excluded if they described just one dimension for HRQoL, such as physical function only. Language restriction was applied in the end stage of search to reduce language selection bias. The languages selected was English or Swedish (when appropriate). The PubMed search strategy is available in Supplemental file 1.

### Study selection

In the second stage, all full articles were evaluated using a predefined data extraction form developed for this study in a Microsoft Access-database, with the following predefined criteria; (1) assessment of HRQoL, (2) exclusion criteria including, (3) demographic characteristics and comparisons of responders vs. non-responders, and (4) HRQoL comparisons adjusted for age and sex.

In the third stage, all the studies identified in the previous stage were selected for review if they met the final predefined inclusion criteria; HRQoL comparisons adjusted for age or comorbidity. The final selection of articles was made by all authors, based on the reporting of all necessary data and in accordance with the predefined inclusion and exclusion criteria. Discrepancies were resolved by consensus.

## Results

### Search results and characteristics of the included studies

A total of 1588 references were identified by the database searches, and in addition one reference was identified through reference lists. After duplicated articles (same findings by different search engines) were removed (n = 1041) 548 abstracts were screened. Of these, 496 articles were excluded after reading the title and the abstract because they did not meet the predefined study criteria. The remaining 52 articles were read in full and of these 41 articles were excluded because they did not meet the final inclusion criteria; HRQoL comparisons adjusted for age or comorbidity. Finally, a total of 11 articles were included in the review (Fig. 1).

Most studies were conducted in Europe [13–19], two in Australia [20, 21], and one each in Canada [22] and USA [23]. All studies were observational prospective cohorts, except one that was a case–control study [21], one a cross-sectional comparison study [15], and one that was a retrospective cohort study (Table 1).

**Table 1:** Characteristics of included studies

References	Country	Study design	HRQoL assessment instrument	Method of HRQoL assessment	Eligible patients for long-term HRQoL assessment, N (%)	Aim of the study
McNelly et al. [13]	United Kingdom	Prospective outcome study	SF-36 version 2	Assessed at a home visit	56 (62)	Explore the relationship between physical activity, HRQoL and clinician-reported frailty. And the relationship between chronic disease status and functional outcome
Williams et al. [21]	Australia	Case–control study	SF-36 version 2, EORTC QLQ-C15-PAL	Face-to-face and telephone interview	No	Describe QoL, symptom profile and health service use after ICU discharge for ICU pat with and without comorbidity
Soliman et al. [18]	The Netherlands	Prospective cohort study	EQ-6D	Mailed questionnaire	4647 (78)	Describe long-term survival and HRQoL for patients after ICU, and for important subgroups
Bagshaw et al. [22]	Canada	Multicentre prospective observational cohort study	EQ-5D (VAS, Index and dimensions), SF-12 (PCS, MCS)	Telephone interview	1359 (62)	Examine the association between frailty and HRQoL among survivors of critical illness
Paparrigopoulos et al. [17]	Greece	Prospective cohort study	SF-36, PCS and MCS or do they mean PF and MH?	Semi-structured telephone interview	48 (16)	Investigate the long-term prevalence of depression and PTSD post-ICU discharge and the impact of ICU hospitalization on the patients HRQoL
Orwelius et al. [15]	Sweden	Cross-sectional comparison	SF-36	Mailed questionnaire	2386 (45)	To test whether stratifying for coexising conditions would reduce differences in HRQoL between ICU-pat and normal controls, and describe those with the lowest HRQoL
van den Boogaard et al. [19]	The Netherlands	Prospective 18-month follow-up study	SF-36	Mailed questionnaire	1361 (84)	Compare the HRQoL including cognitive functioning after ICU discharge for patients with and without delirium during ICU stay, and if different types of delirium exerted different effects of HRQoL
Feemster et al. [23]	USA	Prospective longitudinal multicentre study	SF-36	Mailed questionnaire	18,822 (68)	Determine the association of ICU stay with declines in HRQoL. And to study the effect of critical vs not critical illness on changes i HRQoL
Orwelius et al. [16]	Portugal	Prospective multicentre study	EQ-5D	Mailed questionnaire	935 (80)	Assess the association between memory and HRQoL of patients discharged from the ICU
Oeyen et al. [14]	Belgium	Retrospective based on a 1-year prospective cohort study	EQ-5D	Face-to-face interview at baseline, Mailed questionnaire at 1 year	1867 (96)	Develop an easy to use prediction model for the mean QoL at 1 year after ICU discharge in general critically ill patients based on data at the first ICU day
Haines et al. [20]	Australia	Prospective, observational follow-up study	SF-36 version 2	Questionnaires in-person or by telephone interview	68 (81)	Investigate long-term mortality, physical function, psychological outcomes, and HRQoL in a mixed ICU cohort

*SF-36* Short Form 36 questions; *PCS* Physical Component score; *MCS* Mental Component score; *QLQ-C15-PAL* European Organisation for Research and Treatment of Cancer; *EQ-6D* EuroQol-6 dimension; *SF-12* Short Form 12 questions; *EQ-5D* EuroQol-5 dimensions

### Instrument and methods for measure of HRQoL

HRQoL was assessed using two general tools (SF-36 or SF-12 and EQ-5D) and one palliative care-specific tool (EORTC QLQ-C15-PAL). The most frequently used instrument was the Medical Outcomes Study 36-Item Short-Form health Survey (SF-36) [13, 15, 17, 19–21, 23] or its short-version 12-Item Short-Form Health Survey (SF-12) [22] (62%), followed by the EuroQol-5D (EQ-5D) [14, 16, 18, 22] (31%), whereas the European Organization for Research and Treatment of Cancer (EORTC) QLQ-C15-PAL were used in one study [21] (7%). Two studies used a combination of HRQoL instruments: EORTC QLQ-C15-PAL with the SF-36 [21], and the EQ-5D with the SF-12 [22] (Table 1).

Method of assessment for HRQoL varied between the 11 included studies. Almost half of them (46%) used mailed questionnaires [15, 16, 18, 19, 23], whereas two used telephone interview (18%) [17, 22], two used face-to-face interview (18%) [13, 14], and two used a combination of face-to-face interview and/or telephone interview (18%) [20, 21] (Table 1).

An overview of HRQoL assessment after ICU discharge is shown in Table 2. Inclusion periods were for most of the studies between one and four years [13, 15, 18, 20–23] and ≤ 1 year in two of the studies [16, 19], whereas the inclusion period was not presented in two of the studies [14, 17].

**Table 2:** Assessment of health-related quality of life after ICU

References	Inclusion period	Recruited patient cohort/inclusion criteria	Response rate, N (%) of HRQoL responders	Follow-up period	Control group
McNelly et al. [13]	August 2009–April 2011	91 patients with ICU LoS ≥ 7 days and invasively ventilated ≥ 48 h	27	18 months after ICU-discharge	Norm-based healthy control cohort
Williams et al. [21]	2011–2012	Unknown, ≥ 18 years	60	6 months (or until death if before 6 months)	ICU patients without severe comorbidity
Soliman et al. [18]	July 2009–May 2012	5934 consecutive patients	3034 (65,3)	12 months	Age- and sex-matched general population
Bagshaw et al. [22]	February 2010-July 2011	2180 patients ≥ 50 years and ICU LoS ≥ 24 h	262 (62,2)	6, 12 months after hospital discharge	Normative data (SF-12), Alberta general population (EQ-5D)
Paparrigopoulos et al. [17]	No	308ICU LoS > 24 h	48 (16)	18–24 months after ICU discharge	None
Orwelius et al. [15]	August 2000–June 2004	5306 consecutive patients aged between 20 and 74 years, and ICU LoS > 24 h, and alive 6 months after hospital discharge	780 (32,7)	6 months after hospital discharge	General population stratified for comorbidity/no disease
van den Boogaard et al. [19]	February 2008–February 2009	1613 consecutive patients ICU LoS > 24 h	915 (67,2)	18 months	General population
Feemster et al. [23]	January 1997–August 2000	27,765 consecutive patients visiting the primary care provider at least once in the year prior	11,243 (59,7)	3,6,12,18,24,30 months after enrolment	Hospitalised patients (not ICU), and not hospitalised patients
Orwelius et al. [16]	January 2005–June 2005	1174 consecutive patients ≥ 18 years, and ICU LoS > 48 h, and alive 6 months after ICU discharge	310 (33,5)	6 months after ICU discharge	None
Oeyen et al. [14]	No	1953 consecutive patients ≥ 16 years	1831 (93,8)	12 months	None
Haines et al. [20]	May 2012–December 2013	84 patients > 18 years, and ICU LoS ≥ 5 days	56 (82,3)	4–5 years after ICU-discharge	Age-matched general population

*LoS* Length of stay

A total of 18,566 critically ill patients were included in the 11 studies, and the response rate ranged between 16 and 94%.

### Follow-up period

Follow-up periods for the assessment of HRQoL varied between the included studies from short follow-up (3 months) to long-term follow-up (5 years). All studies had a strict time-point for follow up except for two that had wide ranges of 18–24 months [17] and 4–5 years [20] after ICU discharge, respectively. The strict time points varied between 6 months [15, 16], 12 months [14, 18], and 18 months [13, 19]. Two of the studies assessed HRQoL over time at several occasions: 6 and 12 months after hospital discharge [22], and 3, 6, 12, 18, 24, and 30 months after enrolment [23] (Table 2).

### *Study criteria (*Table 3*)*

**Table 3:** Study specifics

References	Shows detailed HRQoL scores	Exclusion criteria included	Description/comparisonof responders vs. non-responders	HRQoL comparisons adjusted for age and sex	HRQoL comparisons adjusted for comorbidity/ Comorbidity assessment
McNelly et al. [13]	No	Yes; ICU LoS < 7 days, invasively ventilated < 48 h, if pregnant, a lower limb amputee, or suffering a primary neuromuscular pathology or active disseminated cancer	No	Yes	Yes; defined by hospital and general practice coding for management of chronic disease, plus the Charlson co-morbidity index
Williams et al. [21]	No	No	No	No	Yes; immunosuppression, cardiovascular-, renal-, respiratory-, disease, and/or cirrhosis
Soliman et al. [18]	Yes	Yes, readmissions were only included once (the first admission)	No	Yes	Yes; sub grouped by the APACHE IV model as cardiac surgery, sepsis, SAH, traumatic brain injury, others
Bagshaw et al. [22]	Yes/No	Yes; < 50 years and ICU LoS < 24 h, previously enrolled	No	Yes	Yes; by Elixhauser comorbidity score
Paparrigopoulos et al. [17]	No	Yes; ICU LoS < 24 h,	No	No	Yes, from the medical records
Orwelius et al. [15]	Yes	Yes, aged < 20 and > 74 years, ICU LoS < 24 h	Yes comparison of demographic characteristics	Yes	Yes; self-reports of present disease (cancer-, diabetes-, heart failure-, asthma/allergy-, rheumatic-, gastrointestinal-, blood-, kidney-, psychiatric-, or neurologic disease, thyroid or any other metabolic disturbance, or other long-term disease
van den Boogaard et al. [19]	Yes	Yes; ICU LoS < 24 h, sustained coma in ICU, serious auditory or visual disorders, unable to understand Dutch, severe mentally disabled, serious receptive aphasia	Yes comparison of demographic characteristics	Yes	Yes; By APACHE II
Feemster et al. [23]	Yes	No	Yes comparison of demographic characteristics	Yes	Yes; by the Deyo adaption of the Charlson comorbidity index
Orwelius et al. [16]	No	Yes; < 18 years, ICU LoS < 48 h	Yes comparison of demographic characteristics	No	Yes; by the patients physician according to specified categories
Oeyen et al. [14]	No	Yes; < 16 years, readmissions were only included once (the first admission), after cardiac surgery	No	No	Yes; by the Charlson comorbidity index
Haines et al. [20]	Yes	Yes; ≤ 18 years, ICU LoS < 5 days, major disorders affecting the CNS, unable to perform physical outcome measurespre-morbidly	No	Yes	No

*HRQoL* Health-related quality of life; *APACHE* Acute physiology and chronic health evaluation

All the included studies met the third, and final quality criteria stage for inclusion; the HRQoL comparisons adjusted for age or comorbidity. All but one study [20] did HRQoL comparisons adjusted for comorbidity, and more than half of them (54%) did HRQoL comparisons adjusted for both comorbidity, age, and sex [13, 15, 18, 19, 22, 23].

However, only two (18%) of them met all the predefined study criteria at the second stage; assessment of HRQoL, exclusion criteria, including demographic characteristics and comparisons of responders vs. non-responders, and HRQoL comparisons adjusted for age and sex [15, 19].

Six of the studies showed detailed HRQoL scores [15, 18–20, 22, 23], but for one of them [22] only the component scores for SF-12 were given. Exclusion criteria were given in all but two studies [21, 23], and a description of the non-responder group and comparison with patients who responded to the HRQoL survey were given in four of the studies [15, 16, 19, 23] (Table 3).

### Health-related quality of life adjusted for comorbidity

A common finding amongst the studies that fulfilled all the inclusion and none of the exclusion criteria (n = 11), was that comorbidities were found to be the most important factor affecting HRQoL after critical care. Amongst the eleven studies, seven used comorbidity scoring systems (Charlson (n = 7) and APACHE (n = 4)). A significant observation was that three studies (n = 3) included a comorbidity-adjusted control group (Table 2) [15, 21, 23]. For these three studies, there were difficulties in documenting any significant effect of the critical care period itself on the registered HRQoL after the critical care period. A summary of the major findings for long-term HRQoL per article is shown in Table 4.

**Table 4:** Major findings and factors influencing long-term health-related quality of life

References	Comorbidity assessment by	Long-term HRQoL; major finding	HRQoL, Influencing factors
McNelly et al. [13]	By hospital and general practice coding for management of chronic disease, plus the Charlson Comorbidity index	Degree of HRQoL and level of Physical activity are worst for those with pre-admission chronic disease	Pre-admission chronic disease states
Williams et al. [21]	APACHE II	Significant differences within the groups but not between the groups for the patients with or without comorbidity in HRQoL, symptom profile or health service 6 months after ICU discharge	Pat. with chronic disease have reduced HRQoL compared with ICU-pat. previously healthy
Soliman et al. [18]	APACHE IV	One year after ICU admission HRQoL were significantly lower than in the general population except for cardiac surgery patients	Comorbidity pre-ICU Increasing amount of comorbid conditions at ICU admission affected lower survival
Bagshaw et al. [22]	Compared HRQoL with results from critical illness groups in other studies	Frail patients experienced worse HRQoL at 6 (EQ-5D) and 12 months (EQ-5D and (minimal) SF-12 component) compared with not frail patients	Frailty has neg. effect on HRQoL
Paparrigopoulos et al. [17]	Medical records	PTSD and depression share a high degree of comorbidity and persist for a substantial percentage of ICU survivors up to 2 years after ICU discharge which have a negative effect on HRQoL	Lifetime history of psychiatric disease
Orwelius et al. [15]	Self-reports of present disease	After stratifying for comorbidity and adjusting for age and sex, no significant differences in HRQoL were found between ICU patients and the general population. In a subgroup of patients with low HRQoL scores, the mental dimensions were reduced up to 22% and seen in patients with > 1 comorbidity	Co-existing diseases, male sex, marital state single, on sick leave before ICU
van den Boogaard et al. [19]	APACHE II	After adjusting for APACHEII score, sepsis, ICU LoS, gender, and urgent admission no significant differences in HRQoL were found between ICU patients with or without delirium. The HRQoL were worse for the ICU patients on several SF-36 domains compared with the general population	None after adjusting for APACHEII score, sepsis, ICU LoS, gender, and urgent admission
Feemster et al. [23]	The Deyo adaptation of the Charlson comorbidity index, and self-reported diagnoses of depression	No significant difference in the decline in HRQoL between hospitalises patients with or without ICU care	Hospitalization
Orwelius et al. [16]	The patient’s physician	Having memories of the ICU stay is significantly associated with a higher perceived HRQoL compared with the ICU patients having no memories at all	Previous health problems, and less educated
Oeyen et al. [14]	The Charlson comorbidity index	Baseline health status and surgical patients (vs medical) were associated with more positive outcome at 1 year	Baseline health status
Haines et al. [20]	APACHE II	Survivors after critical illness achieved a high level of recovery for physical function and HRQoL with low psychological morbidity comparable with population norms	None

*HRQoL* Health-Related Quality of Life; *EQ-5D* EuroQol-5 dimensions; *SF-12* Short Form-12 questions; *PTSD* Posttraumatic stress disorder; *APACHE II* Acute physiology and chronic health evaluation; *LoS* Length of stay; *SF-36* Short form 36 questions; *PCS* Physical component score; *MCS* Mental component score

## Discussion

**Fig. 2:**
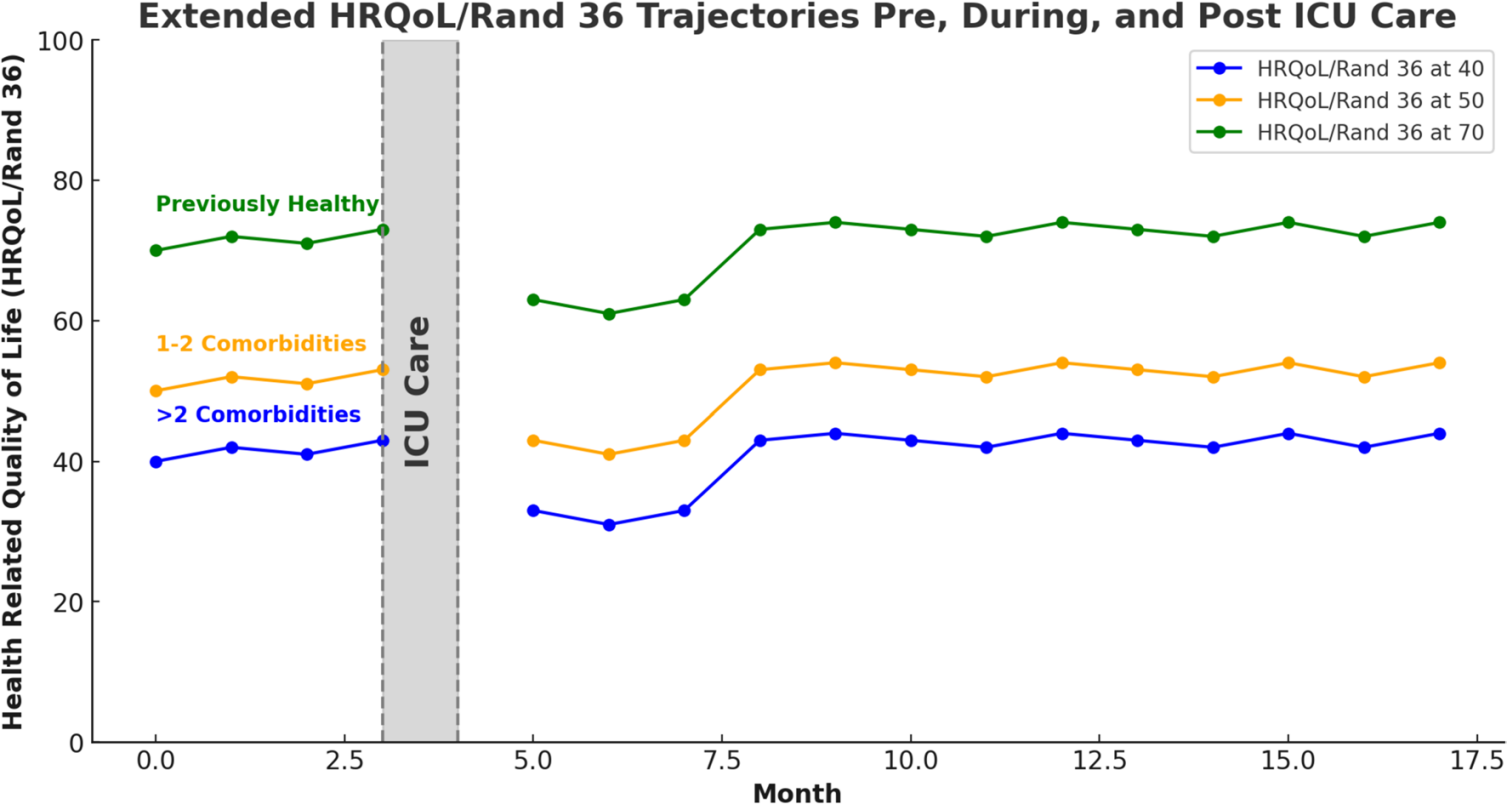
Summary of Health-Related Quality of Life (HRQoL) Effects Post-ICU Care. This review, despite the limited number of underlying studies and their variations, suggests minimal impacts on Health-Related Quality of Life (HRQoL) following ICU care, beyond those typically attributable to comorbidities alone. However, this generalization may occasionally mask significant deterioration of an existing comorbidity or the onset of a newly diagnosed comorbidity. The common early, transient decline in HRQoL typically observed post-ICU care is included. The HRQoL levels depicted in the figure are based on RAND-36/SF-36 protocols. These levels are representative of patients across three categories: those previously healthy, those with 1–2 comorbidities, and those with more than 2 comorbidities. HRQoL: Health-Related Quality of Life; ICU: Intensive Care Unit

The ambition of this purpose-specific review has been to examine how comorbid conditions affect long-term (HRQoL) outcome after critical illness. From a definition-specific perspective, it is obvious that health status alters the HRQoL. However, in the present literature little emphasis is put on comorbidities [24], and consequently it may be difficult to assess the effect of ICU care specifically on HRQoL. The main finding of this review is that when comorbidity is added properly in the modelling of post-ICU HRQoL, it significantly alters the interpretation of HRQoL after intensive care. In the 11 studies included, 7 were adjusted for comorbidities by Charlson score and 4 were by use of APACHE. In all eleven studies looking specifically at comorbidities the comorbidity factor was the most important for the long term HRQoL outcome. In contrast to this effect and finding, the three studies that also included a comorbidity adjusted control group, found an even stronger effect of the comorbidities as the difference between the patient group and the control group completely disappeared and thus stronger and differently supported the comorbidity effect. From a methodological perspective it then seems important to include comorbidity adjusted control groups. A significant shortcoming of the research field shown in this review is the heterogeneity of the studies included. Despite these, an overarching finding is the significant effect of comorbidity acquired before the intensive care period on HRQoL outcome after critical care. As only a fraction of studies of HRQoL after critical care include a comorbidity estimate (See Fig. 1) the present study underlines the need to always include such a measure in future studies of HRQoL after intensive care. Furthermore, a more stringent approach to the protocols used may be asked for as the studies finally selected in this review had large variations in the HRQoL instrument used, the extent of the follow-up time, the comorbidity assessment technique, and the age and sex adjustments (Fig. 2).

## Methodology

### Comorbidity and HRQoL

Most of the comorbidity assessments in the present review used the APACHE score comorbidity alternatives. These have been developed not to adjust for comorbidity effect on HRQoL but to adjust for mortality [25]. A similar problem exists for the mostly commonly used comorbidity instrument, the Charlson index [10]. Also, this index is based not on the outcome measure HRQoL, but the effect of comorbidities on mortality. This shortcoming is well known, and an alternative adjustment tool is the Mukherjee index that has been developed to adjust HRQoL outcome for comorbidity [26]. However, this index has not yet, to our knowledge, been applied in the critical care setting.

In the studies selected for this review, three papers tried to construct a comorbidity adjusted control group [15, 21, 23]. Interestingly, in these papers no significant effects of the critical care period itself could be linked to HRQoL after the intensive care period. In the present review we chose not to include smaller subgroup studies. However, it needs to be mentioned that there are studies that have investigated a specific diagnosis treated in ICU [27–29]. In such a study of chronic obstructive pulmonary disease (COPD) [30], a control group with the same COPD score, but not treated in the ICU, was compared with COPD patients treated in the ICU. The interesting finding was that the authors could not find a specific HRQoL effect of the ICU treatment period itself. This supports the findings in the above-cited three studies which included comorbidity adjusted control groups.

### Comorbidity, age, and sex

In many studies, adjustments for HRQoL in the intensive care cohorts are made based on age. This is of course important, as we know there are age-related effects on HRQoL [31]. Furthermore, age adjustment has been claimed to be important to compensate for comorbidities as comorbidities increase with age [31]. However, it has repeatedly been shown that the cohorts that end up in the ICU are different compared to the general population in the rate of comorbidities, irrespective of age [9, 32]. Therefore, it needs to be pointed out that age is not specific enough for such an adjustment. Furthermore, sex adjustments are often made for the same reasons. This can make sense as it is known that there are sex-related differences in the selection, treatment, and outcome process of the ICU cohorts [33, 34]. However, it may not fully compensate for more detailed information about comorbidities.

### Limitations

The major limitation of this review resides in the scant retrieval of studies reported. Furthermore, in the studies selected there was a large heterogeneity in important study related parameters, i.e., HRQoL instruments, comorbidity, age, and sex adjustments as well as follow-up period. This significantly reduces the strength of the conclusions made. However, for the three studies that included comorbidity adjusted control groups the findings were coherent, supporting the overall conclusion of our review [15, 21, 23].

### Future aspects

This review underlines the need for proper adjustments of the comorbidity effects, as lacking comorbidity adjustments may reduce the validity of the HRQoL studies made in ICU populations. The effect of comorbidity is large, and the ICU population in most high-income countries is more significantly burdened by comorbidities the more they age. To properly adjust for this is essential. HRQoL data in individual patients prior to intensive care is rare. Further, prospective pre-ICU HRQoL measured by the patient or proxies after the intensive care period is at risk of bias and should be used with caution [35, 36]. Therefore, trying to construct an appropriate comorbidity adjusted control group appears to be the method of choice. If comorbidity adjustments are planned, it is important to use an HRQoL instrument that is comorbidity validated [26] rather than a mortality related one. In addition, age and sex adjustments are important as both affect HRQoL. From the present study it seems reasonable to suggest SF-36 as the HRQoL instrument, as it better covers HRQoL issues than e.g., EQ-5D. The appropriate follow-up time is more difficult to suggest. One alternative is to extend it beyond 6–12 months, when many patients are still recovering from their critical illness, and to also extend the time to at least three years so that observations can be gathered in a stable period [9].

## Conclusions

In summary, comorbidity is a main factor affecting the patients self-perceived Health-Related Quality of Life (HRQoL) in individuals who have been in the ICU. A major challenge in this area of study is the considerable diversity in the methodologies of the reviewed research, especially regarding the tools used for HRQoL assessment, the follow-up period, the approach to evaluating comorbidity, and considerations for age and gender. Despite these methodological differences, the predominant finding suggests that comorbidity alone is responsible for most of the observed HRQoL changes in the critical care survivor population. The present lack of thorough research in this area highlights the increasing necessity for more expansive and detailed studies, particularly those that incorporate comorbidity into the final HRQoL evaluation.

### Supplementary Information

Supplementary Material 1.

Supplementary Material 2.

## Data Availability

Not applicable.

## Abbreviations

APACHE: Acute physiology and chronic health evaluation
COPD: Chronic obstructive pulmonary disease
EORTC QLO-C15-PAL: European Organization for Research and Treatment of Cancer
EQ-5D: EuroQol 5 dimensions
ICU: Intensive care unit
HRQoL: Health related quality of life
PICO: Population, intervention controls outcome
PRISMA: Preferred reporting items for systematic reviews and meta-analyses
SAPS3: Simplified acute physiology score 3
SF-12: Short form 12 questions
SF-36: Short form 36 questions
