# Supplementary material for: Is comorbidity alone responsible for changes in health-related quality of life among critical care survivors? A purpose-specific review
Source: Crit Care. 2024 Jun 26;28:208. doi: 10.1186/s13054-024-04997-x (PMC11201873; doi:10.1186/s13054-024-04997-x)
Supplement: Supplementary file 1 — Supplementary Material 1. [file 13054_2024_4997_MOESM1_ESM.docx]

**Supplementary file 1**

Search string Pubmed

#8,"Search (intensive care unit OR critical care OR critical illnessOR ICU) AND (follow-up OR aftercare) AND (health-related quality of life OR HRQoL OR QoL) AND (ADL OR Mortality) Filters: Publication date from 2010/01/01 to 2021/12/31, adult, English (language)

#7,"Search (intensive care unit OR critical care OR critical illnessOR ICU) AND (follow-up OR aftercare) AND (health-related quality of life OR HRQoL OR QoL) AND (cognitive dysfunction) Filters: Publication date from 2010/01/01 to 2021/12/31, adult, English (language)

#6,"Search (intensive care unit OR critical care OR critical illnessOR ICU) AND ((follow-up OR aftercare) AND (health-related quality of life OR HRQoL OR QoL) AND (physical ability) Filters: Publication date from 2010/01/01 to 2021/12/31, adult, English (language)

#5,"Search (intensive care unit OR critical care OR critical illnessOR ICU) AND (follow-up OR aftercare) AND (health-related quality of life OR HRQoL OR QoL) Filters: Publication date from 2010/01/01 to 2021/12/31, adult, English (language)

#4,"Search (intensive care unit OR critical care OR critical illness OR ICU) AND (follow-up OR aftercare) AND (health-related quality of life OR HRQoL OR QoL)” Filters: Publication date from 2010/01/01 to 2021/12/31

#3,"Search (intensive care unit OR critical care OR critical illness OR ICU) AND (follow-up OR aftercare)” Filters: Publication date from 2010/01/01 to 2021/12/31

#2,"Search (intensive care unit OR critical care OR critical illness OR ICU)” Filters: Publication date from 2010/01/01 to 2021/12/31

#1,"Search (intensive care unit OR critical care OR critical illness OR ICU)”
