# Supplementary material for: Is comorbidity alone responsible for changes in health-related quality of life among critical care survivors? A purpose-specific review
Source: Crit Care. 2024 Jun 26;28:208. doi: 10.1186/s13054-024-04997-x (PMC11201873; doi:10.1186/s13054-024-04997-x)
Supplement: Supplementary file 2 — Supplementary Material 2. [file 13054_2024_4997_MOESM2_ESM.docx]

| **Supplementary file 2** Excluded studies after the full-text screening | | | |  |
| --- | --- | --- | --- | --- |
| **Author** | **Year** | **Country** | **Title** | **Reason for exclusion** |
| Aitken | 2015 | Australia | Physical recovery in intensive care unit survivors | HRQoL comparisons not adjusted for age and sex |
| Calvo-Ayala | 2013 | USA | Interventions to improve the physical function of ICU survivors | Focus on physical function only |
| Capuzzo | 2015 | Italy | Our paper 20 years later: 1 year survival and 6-months quality of life after intensive care | HRQoL comparisons not adjusted for age and sex |
| Chahraoui | 2015 | France | Psychological experiences of patients 3 months after a stay in the ICU | Not HRQoL |
| Clancy | 2015 | UK | The psychological and neurocognitive consequences of critical illness | Not HRQoL |
| Cuthbertson | 2010 | Canada | Quality of life in the five years after intensive care | Excludes all ICU patients <Level 3 ICU |
| Das Neves | 2015 | Argentina | Symptom burden and health-related quality of life among intensive care unit survivors i Argentina | HRQoL comparisons not adjusted for age and sex |
| De Franca | 2012 | Brazil | Physical therapy in critically ill adult patients | Not HRQoL |
| Denehy | 2013 | Australia | Exercise rehabilitation for patients with critical illness | HRQoL comparisons not adjusted for age and sex |
| Dennis | 2011 | Australia | How do Australian ICU survivors fare functionally 6 months after admission? | Not HRQoL |
| Dettling-Ihnenfeldt | 2016 | The Netherlands | Coping style and quality of life in Dutch intensive care unit survivors | Excluded patients <72 hrs in ventilator. HRQoL comparisons not adjusted for age and sex |
| Elliott | 2011 | Australia | HRQoL and physical recovery after a critical illness | Excluded patients with ventilator <24h |
| Farley | 2016 | Australia | A feasibility study of functional status and follow-up clinic preferences of patients at high risk of PICS | Excluded patients with <7 days ventilation. HRQoL comparisons not adjusted for age and sex |
| Griffiths | 2013 | UK | An exploration of social and economic outcome and associated with HRQoL after critical illness in general ICU survivors | Not HRQoL |
| Haas | 2013 | Brazil | Factors influencing physical functional status in ICU survivors two years after discharge | Not HRQoL |
| Hatchett | 2010 | South Africa | psychological sequelae following ICU admission at a level 1 academic south Africa hospital | Not HRQoL |
| Hofhuis | 2015 | The Netherlands | ICU survivors show no decline in health-related quality of life after 5 years | HRQoL comparisons not adjusted for age and sex |
| Hough | 2013 | USA | Improving physical function during and after critical care | Focus on physical function only |
| Johns | 2010 | UK | Considerations and proposals for the management of patients after prolonged ICU admission | Not HRQoL |
| Jones | 2010 | UK | Intensive care diaries reduce onset PTSD following critical illness | Not HRQoL |
| Kayambu | 2013 | UK | Physical therapy for the critically ill in the ICU | Not HRQoL |
| Kelly | 2010 | Australia | Patients’ recovery after critical illness at early follow-up | Focus on physical function only |
| Lim | 2016 | UK | Conceptualizing and measuring health-related quality of life in critical care | HRQoL comparisons not adjusted for age and sex |
| Mafra | 2016 | Brazil | Quality of life of critically ill patients in a developing country | HRQoL comparisons not adjusted for age and sex |
| McKinley | 2016 | Australia | Health-related quality of life and associated factors in intensive care unit survivors 6 months after discharge | HRQoL comparisons not adjusted for age and sex |
| McWilliams | 2016 | UK | Outpatient-based physical rehabilitation for survivors of prolonged critical illness | Excluded patients with ventilator <5 days |
| Myhren | 2010 | Sweden | HRQoL and return to work after critical illness in general ICU patients | HRQoL comparisons not adjusted for age and sex |
| Orsini | 2015 | USA | Prognostic factors associated with adverse outcome among critically ill elderly patients admitted to the ICU | Focus on older ICU patients |
| Orwelius | 2011 | Sweden | Social integration an important factor for HRQoL after critical illness | Examine the impact of social integration for HRQoL after ICU |
| Quasim | 2015 | UK | Employment, social dependency and return to work after intensive care | HRQoL comparisons not adjusted for age and sex |
| Rommes | 2013 | The Netherlands | The life of ICU-survivors | Not HRQoL |
| Schenk | 2012 | Australia | Health-related quality of life of long-term survivors of intensive care | HRQoL comparisons not adjusted for age and sex |
| Sidiras | 2019 | Greece | Long term follow-up of quality of life and functional ability in patients with ICU acquired weakness | HRQoL comparisons not adjusted for age and sex |
| Solverson | 2016 | Canada | Assessment and predictors of physical functioning post-hospital discharge in survivors of critical illness | HRQoL comparisons not adjusted for age and sex |
| Steenbergen | 2015 | Denmark | intensive care delirium - effect of memories and health-related quality of life | HRQoL comparisons not adjusted for age and sex |
| Svenningsen | 2014 | Denmark | Intensive care delirium - effect of memories and health-related quality of life | Data not adjusted for comorbidity, age, or sex |
| Vesz | 2013 | Brazil | Functional and psychological features immediately after discharge from an ICU | Not HRQoL |
| Wade D | 2012 | UK | Investigation risk factors for psychological morbidity three months after intensive care | HRQoL comparisons not adjusted for age and sex |
| Walsh TS | 2015 | Scotland | Increased hospital-based physical rehabilitation and information provision after ICU discharge | HRQoL comparisons not adjusted for age and sex |
| Wolters AE | 2014 | The Netherlands | Long-term outcome of delirium during ICU stays in survivors of critical illness | HRQoL comparisons not adjusted for age and sex |
| Zetterlund P | 2012 | Sweden | Memories from ICU persist for several years | HRQoL comparisons not adjusted for age and sex |

HRQoL; health-related quality of life
